# *Legionella longbeachae* and Legionellosis

**DOI:** 10.3201/eid1704.100446

**Published:** 2011-04

**Authors:** Harriet Whiley, Richard Bentham

**Affiliations:** Author affiliation: Flinders University, Adelaide, South Australia, Australia

**Keywords:** Legionella longbeachae, potting mix, Legionnaires’ disease, bacteria, Pontiac fever, synopsis

## Abstract

Reported cases of legionellosis attributable to *Legionella longbeachae* infection have increased worldwide. In Australia and New Zealand, *L. longbeachae* has been a known cause of legionellosis since the late 1980s. All cases for which a source was confirmed were associated with potting mixes and composts. Unlike the situation with other *Legionella* spp., *L. longbeachae*–contaminated water systems in the built environment that cause disease have not been reported. Spatially and temporally linked outbreaks of legionellosis associated with this organism also have not been reported. Sporadic cases of disease seem to be limited to persons who have had direct contact with potting soil or compost. Long-distance travel of the organism resulting in infection has not been reported. These factors indicate emergence of an agent of legionellosis that differs in etiology from other species and possibly in route of disease transmission.

*Legionella* spp. were first identified as organisms of public health significance in 1976 and are now recognized as the causative agent of legionellosis. *L. pneumophila* was the species responsible for this initial disease outbreak and has remained the major cause of legionellosis (*1**,**2*). The clinical manifestations of legionellosis range from no symptoms to acute atypical pneumonia and multisystem disease (*2*). The term legionellosis refers collectively to the clinical syndromes resulting from *Legionella* spp. infection, i.e., Legionnaires’ disease (a *Legionella* spp.–derived pneumonic infection) and Pontiac fever (an acute, self-limited febrile illness that has been linked serologically and by culture to *Legionella* spp.) (*1**,**2*). Community- and hospital-acquired legionelloses typically are associated with water systems in the built environment, such as cooling towers, spas, showers, and other warm water systems (*1**,**2*). Protozoa play a major role in the multiplication and dissemination of *Legionella* spp. in natural environments. The parasitism of amoebae and ciliates is well documented, and this parasitic capability is the basis of human disease through infection of human lung macrophages (*1**,**2*).

*L. longbeachae* was first isolated in 1980 from a patient with pneumonia in Long Beach, California, USA (*3*). A second serogroup of *L. longbeachae* was discovered during the same year (*4*). Neither of these reports suggested a recognized source of infection.

In Europe, *L. pneumophila* is responsible for 95% of cases of Legionnaires’ disease. Of the remaining 5%, the most common causative agent is *L. longbeachae* (*5*). In Australia, New Zealand, and Japan, reported cases of *L. longbeachae* infection occur as often as cases of *L. pneumophila* infection (*6**–**8*). Within the past decade, the number of *L. longbeachae* reports has increased markedly across Europe and parts of Asia (*9**–**15*).

## Potting Mixes

*L. pneumophila* is primarily aquatic and endemic to warm water in the built environment (e.g., cooling towers, shower heads, and water fountains) and in natural environments (e.g., rivers and lakes) (*1**,**2*). It is transmitted from the environment through inhalation of aerosol or aspiration of *Legionella* spp.–contaminated particles (*1**,**2*). *L. longbeachae* is rarely isolated from aquatic environments (*16**,**17*). The primary environmental reservoir of *L. longbeachae* remains unknown; however, the major source of human infection is considered to be commercial potting mixes and other decomposing materials, such as bark and sawdust (*5**,**8**,**18**,**19*). No reports of *L. longbeachae* infection from water systems in the built environment have been confirmed.

Recent analysis of the *L. longbeachae* genome has demonstrated that it is highly adapted to the soil environment. The genome encodes for a range of proteins that might assist in the invasion and degradation of plant material (*20*). These enzyme systems are not present in *L. pneumophila*. This work supports the hypothesis of a possible environmental association with certain plant species (*8**,**18*).

The link between potting mix and legionellosis was established in 1989 when a cluster of *L. longbeachae* infections was detected in South Australia. Investigations identified commercial potting mixes as the source of disease (*18*). Since then, *L. longbeachae* commonly has been isolated from fresh potting mixes and some of its components but less commonly from natural soils, which suggests that the composting process may be a catalyst for growth. The heat and high moisture content during composting may allow for multiplication of several *Legionella* species to detectable levels (*18*). The route of transmission of *L. longbeachae* from contaminated environmental samples remains unknown (*7**,**18*).

In 1990, a study determining the incidence of *Legionella* spp. in potting mix found that more than two thirds (33/45) of Australian potting mixes and none (0/19) of European potting mixes tested positive for *Legionella* spp (*18*). The authors postulated that the discrepancy between incidence of *L. longbeachae* infection in Australia and the rest of the world, particularly Europe, was attributable to the content of commercial potting mix. In Australia, potting mix is made mostly from composted pine waste products, such as sawdust and hammer-milled bark. In Europe, peat is the main component of potting mix (*16**,**18*). In 2001, a similar study in Japan found that 2 of 24 commercial potting mixes contained *L. longbeachae.* The main component of Japanese potting mix is composted wood products, particularly composted oak. The Japanese study also found that an amoebic enrichment of the potting mixes resulted in 9 of 24 potting mixes testing positive for *L. longbeachae*. This finding demonstrated that *L. longbeachae* can parasitize soil protozoa and that it was present in potting mixes but at numbers lower than the limit detected by using culture (*8*). Genomic analysis subsequently confirmed this parasitic capability (*20*). In 2008, testing for *Legionella* spp. was conducted on 46 commercial potting mixes in Switzerland. Two of 46 were culture positive for *L. longbeachae* and almost half (21/46) for *Legionella* spp. Most (41/46) of the potting mixes tested positive by quantitative PCR for *Legionella* spp. Two thirds of these potting mixes contained peat as the base component. This result contradicted previous studies on European potting mixes but supported the emerging trend of increasing numbers of reported *L. longbeachae* cases (*12**,**18*).

## Detection

Detection of *Legionella* spp. by culture techniques is insensitive. Overgrowth of culture media with competing flora is a major problem (*1**,**2*). This problem is heightened for detection of *L. longbeachae* in potting mixes. Potting mixes have a high microbial load and contain spore-forming bacteria and fungi associated with composting. As a result, heat pretreatment of potting mixes tends to stimulate germination of spores and rapid overgrowth of the agar medium, rather than reduce competing flora. Acid pretreatment is the preferred option (*18*). The variable nature, pH, buffering capacity, and humic content of commercial compost and potting mixes means that the duration of acid pretreatment is best tailored to the individual sample rather than being generically applied (*12**,**18*).

Molecular methods (quantitative PCR) have been used recently to quantitatively detect *Legionella* spp. in potting mixes when culture methods gave negative results (*11*). Improved but nonquantifiable detection in potting soils also have been reported after amoebic enrichment of soil samples (*8*).

## Disease Prevalence

Clinical presentations of *L. longbeachae* infections are similar to those of other legionelloses (*21*). Risk factors for infection in common with other *Legionella* infections are smoking, preexisting medical conditions, and immunosuppression. Gardening activities and use of potting mixes are risk factors that are so far unique to *L. longbeachae* infection (*7*). The disease predominantly affects persons <50 years of age, and reports suggest the median age for infection is slightly higher for *L. longbeachae* than for *L. pneumophila* (*2**,**7**,**16**,**21*). In addition, fewer deaths tend to be associated with *L. longbeachae* infection than with *L. pneumophila* (*21*). The virulence factors associated with *L. longbeachae* clearly differ from those of *L. pneumophila*, which may help explain the differences in disease prevalence and severity (*20*).

Recently, *L. longbeachae*–derived Legionnaires’ disease has increased worldwide. In the Netherlands during 2000–2004, the first 5 reported cases of *L. longbeachae*–derived pneumonia were reported (*13*). Potting mix was associated with infection when analysis found a genotypically identical strain of *L. longbeachae* in the patient’s sputum and in the potting mix. Two other patients of the 5 had indistinguishable genotypes, 1 of whom had visited the same gardening center as the index patient. Unfortunately, further analysis of the cluster was not possible because 3 of the patients died after hospital admission (*13*).

In Thailand, a population-based survey was conducted during 2003–2004 on 556 pneumonia patients >18 years of age who received chest radiographs and etiologic testing. This study found no positive cases of *L. pneumophila* and 20 (5%) cases of *L. longbeachae.* The global increase in infection rates is associated with soils and potting mixes This study did not identify an environmental source of infection (*10*). In 2004, a 25-year-old woman in Spain who had systemic lupus erythematosus died of community-acquired *L. longbeachae*–derived pneumonia (*14*).

During 2008–2009, Scotland recorded a cluster of Legionnaires’ disease caused by *L. longbeachae*. Potting mix was associated with all 3 cases of infection. *L. longbeachae* isolates from patients and potting mix were genotyped by amplified fragment-length polymorphism. The genotypes isolated from the first 2 patients matched the genotypes from the associated potting mixes. No isolate was available from the third patient, but the genotype from the potting mix matched the genotype from the first patient. The first 2 patients had contact with the same brand of potting mix, which contained composted green waste (heat treated at 65°C for 5–10 days) and 30%–50% peat that had not been heat treated. The second patient also had contact with a second brand of potting mix that contained 75%–80% peat that had not been heat treated. The third patient had contact with compost made from expanded wood fiber, coir, and bark (*22*).

These reports contrast with previous reports of *L. longbeachae* in Europe. In 1999, the European Working Group on Legionella Infections reported only 2 cases of *L. longbeachae* from a total of 337 (<1%) reported *Legionella* spp. infections (*22*). In 2008, *L. longbeachae* was noted as the dominant species among non–*L. pneumophila* infections in Europe (*23*).

The number of reported *L. longbeachae* cases might not truly represent the total numbers because the infection in many patients might go undiagnosed. Standard routine diagnostic testing for pneumonia patients involves a legionellosis urine antigen test, which detects only *L. pneumophila* serogroup 1 (*22*). Also, many patients with Pontiac fever might not require hospitalization and might not be aware they have a *Legionella* spp. infection (*1*).

## Survival in the Environment

The mechanisms that enable *Legionella* spp. to infect protozoa also enable opportunistic infection of the alveolar macrophages within human lungs. *Legionella* spp. infect and multiply within protozoan hosts in the absence of any other supporting nutrients (*2*). The relationship between *L. pneumophila* and a range of protozoan hosts has been documented in detail (*1**,**2*). The relationship between *L. longbeachae* and protozoan hosts is not as well understood.

Experimentally, both *L. pneumophila* and *L. longbeachae* infected the ciliate *Tetrahymena pyriformis*, although protozoan susceptibility to infection varied according to strain differences and available nutrients (*24*). In addition, although in situ *L. pneumophila* can infect and multiply within *Acanthamoeba castellanii*, *L. longbeachae* is unable to do so (*25*). Recently both *L. pneumophila* and *L. longbeachae* have been shown to colonize and persist within the intestinal tracts of *Caenorhabditis* nematodes in laboratory assays and soil environments. *Legionella* spp. replicated within the intestinal tract but did not invade surrounding tissue and were excreted as differentiated forms similar in structure to protozoan cysts. This study suggested that nematodes may serve as natural hosts for *Legionella* spp. and assist in their propagation throughout soil environments. The ability of *L. longbeachae* to infect protozoan and metazoan hosts allows for long-term contamination of environmental sites (*26*). The ability to survive protozoan cyst formation might also explain ability of *L. longbeachae* to endure the composting process and survive in desiccated potting mixes (*16**,**18*).

## Disease Transmission

Spatially and temporally linked Legionnaires’ disease outbreaks associated with *L. longbeachae* have never been confirmed. The first cluster of cases detected in South Australia was reported as seasonally but not geographically related (*27*). Seasonal clustering of cases during spring and autumn has been noted in Australia and overseas (*22**,**27*).

Cases of disease typically are sporadic and statistically associated with potting mix use and gardening activities (*28**,**29*). The route of disease transmission remains uncertain, although close proximity or direct contact with composts and potting mixes support hand-to-mouth, aspiration, or aerosolization routes of infection (*7*). No reports have been published that detail infection associated with long-distance travel of *L. longbeachae*, which contrasts markedly with the considerable distances traveled by other *Legionella* spp. during disease outbreaks (*2*).

A recent report detailed an outbreak of *L. longbeachae* infection in a commercial nursery (*28*). In this instance, Pontiac fever was the clinical presentation. Workers were in an enclosed facility without respiratory protection and with considerable potential for dust and aerosol generation. This is the first report of either Pontiac fever or a temporally and spatially confirmed outbreak of legionellosis associated with *L. longbeachae* (*28*).

Reported cases of infection in Asia, Europe, and the United States follow a similar pattern of sporadic disease linked to direct exposure to potting mix and compost (*9**,**10**,**13**,**22**,**29*). The rarity of outbreaks of disease and prerequisite for direct exposure suggest an alternative route of transmission of disease to other *Legionella* spp., and the literature alludes to this information (*7**,**18*). Concentrations of the organism per gram of potting mix have been reported that are comparable to those associated with Legionnaires’ disease per milliliter attributed to water systems (*1**,**2**,**12*). In addition, other disease-causing legionellae are present in potting mixes (*8**,**18*). In only 1 instance has potting mix been (inconclusively) implicated as a possible source of Legionnaires’ disease from an organism other than *L. longbeachae* (*30*). Why potting mix is a source of infection from only this species remains a mystery.

Currently, no strategies are available to control or eliminate *Legionella* spp. in potting mixes. Awareness of health risks associated with handling compost and potting mixes protects against disease; the precise nature of this protective effect is unknown (*7*). In Australia, all bagged potting mixes and compost carry a health warning and recommendations for how to avoid infection. These recommendations include using a face mask, avoiding inhalation of dust and aerosols, and washing hands after using the material (*31*).

## Conclusions

*L. longbeachae* infections have accounted for a major proportion of legionelloses in Australia and New Zealand since the late 1980s (*7*). Recently, the global incidence of reported *L. longbeachae* infections has increased (*9**,**23**,**28*). Factors explaining this emergence of infections are unknown but may be result in part from improved surveillance (*23*). In all reports, disease transmission is associated with soils, composts, and potting mixes rather than with water systems, with which other *Legionella* spp. infections are associated (7*,18.27*). The mechanism of *L. longbeachae* transmission remains unknown, but close association with contaminated material is a recurrent theme (*7*). Long-distance travel of the organism and subsequent infection has not been documented, which may suggest that disease is not transmitted through aerosol inhalation (*7**,**18**,**27*). The environmental reservoir for this *Legionella* species is yet to be identified, and association with a range of plant materials has been postulated (*7**,**18**,**20*). Isolation from peat-based potting mixes confounds this theory to some extent (*12**,**13*). Control strategies for this emerging disease are limited to published warnings on bagged products relating to handling and exposure (*7**,**22**,**31*).
